# Incidence of *Fusarium* Species and Mycotoxins in Silage Maize

**DOI:** 10.3390/toxins3080949

**Published:** 2011-08-04

**Authors:** Sonja Eckard, Felix E. Wettstein, Hans-Rudolf Forrer, Susanne Vogelgsang

**Affiliations:** Agroscope Reckenholz-Tänikon Research Station/Reckenholzstrasse 191, CH-8046 Zürich, Switzerland; Email: sonja.eckard@art.admin.ch (S.E.); felix.wettstein@art.admin.ch (F.E.W); hans-rudolf.forrer@art.admin.ch (H.-R.F.)

**Keywords:** silage maize, cropping factor, prediction, deoxynivalenol, animal feed

## Abstract

Maize is frequently infected by the *Fusarium* species producing mycotoxins. Numerous investigations have focused on grain maize, but little is known about the *Fusarium* species in the entire plant used for silage. Furthermore, mycotoxins persist during the ensiling process and thus endanger feed safety. In the current study, we analyzed 20 Swiss silage maize samples from growers’ fields for the incidence of *Fusarium* species and mycotoxins. The species spectrum was analyzed morphologically and mycotoxins were measured by LC-MS/MS. A pre-harvest visual disease rating showed few disease symptoms. In contrast, the infection rate of two-thirds of the harvest samples ranged from 25 to 75% and twelve different *Fusarium* species were isolated. The prevailing species were *F. sporotrichioides*, *F. verticillioides* and *F. graminearum*. No infection specificity for certain plant parts was observed. The trichothecene deoxynivalenol (DON) was found in each sample (ranging from 780 to 2990 µg kg^−1^). Other toxins detected in descending order were zearalenone, further trichothecenes (nivalenol, HT-2 and T-2 toxin, acetylated DON) and fumonisins. A generalized linear regression model containing the three cropping factors harvest date, pre-precrop and seed treatment was established, to explain DON contamination of silage maize. Based on these findings, we suggest a European-wide survey on silage maize.

## 1. Introduction

Maize silage is an important animal feed, which can be infected by a broad range of toxigenic fungi. Beside storage fungi, such as the aflatoxin producing *Aspergillus fumigatus*,field fungi infecting and producing mycotoxins before harvest, represent a hazard to feed safety [1]. Fungi of the genus *Fusarium* are important pathogens leading to considerable yield losses [2,3]. In small-grain cereals they cause Fusarium head blight (FHB) and in maize stalk and ear rot. Surveys of maize silage revealed that the amount of *Fusarium* fungi could be reduced by the ensiling process [4,5], possibly due to the acidic and anaerobic conditions. In contrast, *Fusarium* mycotoxins, produced before ensiling, are highly stable substances and usually are not affected by ensiling [6,7]. To date, most investigations have focused on toxin content in maize kernels [3,8] and toxins in maize silage [4,9]. However, little is known about the infection of maize plants with *Fusarium* fungi before ensiling or whether these fungi are more specific to certain plant organs [8,10,11].

Compared with small-grain cereals, which are mainly infected by *F. graminearum *(*sensu lato*), *F. avenaceum*, *F. culmorum*, *F. poae* and *F. crookwellense* [2,12], maize is often colonized by a substantially greater number of *Fusarium* species [3,8,11,13]. For instance, 13 different species were found in Swiss maize kernels in 2005 and 15 different species in 2006 [8]. A similar diversity was detected in stalk pieces in 2006 [8]. The great diversity of *Fusarium* species on maize plants suggests the occurrence of inter-species interactions [14]. Additionally, maize is often highly infected and its silage is frequently contaminated by *Fusarium* mycotoxins such as trichothecenes, especially deoxynivalenol (DON), but also zearalenone (ZON) and fumonisins (FUM) (e.g., [3]). Deoxynivalenol can be produced by *F. graminearum*, *F. crookwellense* and *F. culmorum *(cited in [15]). These species and additionally *F. equiseti* and some strains of *F. oxysporum* are also potential ZON-producers [15]. Fumonisins are produced by the typical maize pathogens *F. verticillioides *(synonym *F. moniliforme*), *F. proliferatum*, *F. oxysporum* and some strains of *F. subglutinans* [15]. The European Commission passed threshold values for DON, ZON and FUM in unprocessed cereals and food [16,17] and guidance values for animal feed which also apply in Switzerland [18]. Maize-based animal feed raw materials should not exceed concentrations of DON, ZON and FUM with 12, 3 and 60 mg kg^−1^, respectively. However, guidance values vary depending on the animal species, age of animal as well as use or processing of the animal feed. Some *Fusarium* mycotoxins where shown to be transmitted into animal products, as shown for ZON in meat [19] and several mycotoxins in milk [20,21]. However, as amounts were small, human health effects might be negligible [21].

Several cropping factors influencing fungal growth and mycotoxin contamination of wheat [22] and maize [11,23,24] have recently been studied. A maize-wheat crop rotation and reduced tillage were identified as risk factors for *F. graminearum* infection and elevated DON contamination of wheat, since maize residues served as overwintering substrate [22,25,26]. As a consequence, a modified crop rotation and acceleration of decomposition of maize residues has been urgently recommended [22,25,26]. 

The aim of this study was to investigate (1) the natural *Fusarium* species occurrence and mycotoxin contamination of silage maize from various sites in the Swiss canton Aargau, (2) whether visual disease assessment at harvest would predict *Fusarium* amount and (3) which cropping factors potentially influence infection by toxigenic *Fusarium* species. 

## 2. Materials and Methods

### 2.1. Sampling

A pre-harvest disease rating was conducted on 22 fields. Thereof, 19 harvest samples were obtained for toxin measurements, but morphological-based analyses were conducted from only 17, because one sample was mature silage and another did not lead to *Fusarium* colonies. 

**Figure 1:**
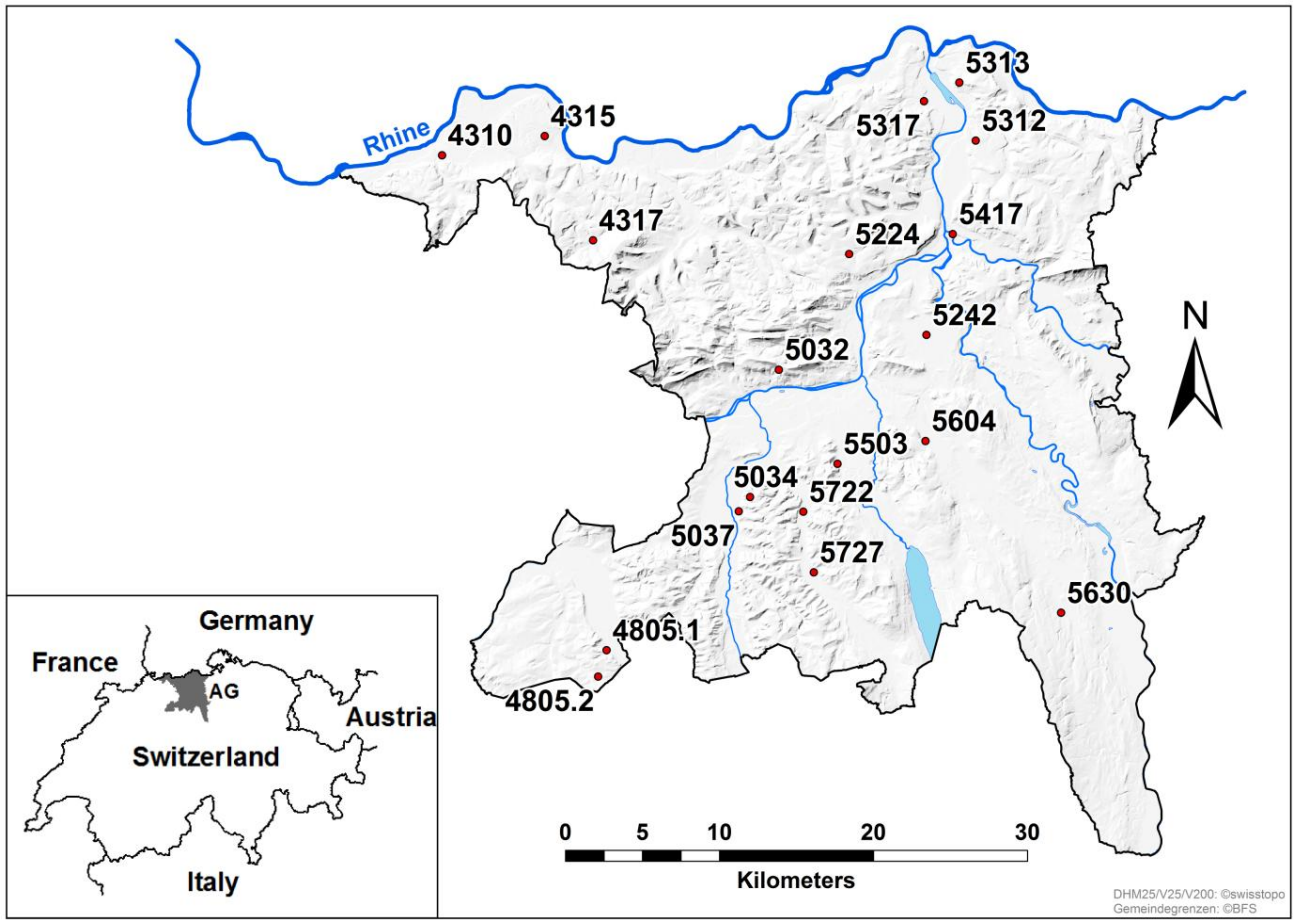
Location of the 19 fields in the Swiss canton Aargau for collection of harvested silage maize. Numbers indicate the postal codes and were used as sample numbers. Map design with ArcMap9.3 [28] by B. Held, ART.

From the same fields, nineteen silage samples from agricultural fields were obtained from different sites in the Swiss canton of Aargau (Figure 1). Samples were obtained by collecting approximately 1 kg of chopped maize silage at ten different positions of the harvested material before ensiling, mixing it carefully and taking a subsample of approximately 2 kg. Information about the cropping techniques applied by the grower was obtained using a questionnaire. The sample was packed in a perforated plastic bag and sent by post overnight. One to 2 days (d) after harvest, samples were dried in the perforated plastic bag in a warm air stream of 32 ± 1 °C for 3 to 4 days and finally stored at 10 °C in the dark. In order to stop fungal growth, while keeping the fusaria viable, these drying conditions were chosen according to previous findings, that *Fusarium* species in maize kernels with a minimum moisture content of 20% grew at 30 °C or below, but were no longer viable at 37 °C [27].

### 2.2. Pre-Harvest Disease Rating

A visual disease rating of maize ears and stalks was conducted to predict a potential infection by toxigenic *Fusarium* species. Because harvest dates were not known in advance, the disease rating was conducted up to 7 weeks before harvest. For this, 50 plants per field were randomly sampled. Stalks were cut in a lateral direction between aerial roots and the main ear. Husk leaves of the main ear were removed. Rot, which was presumably not caused by *Fusarium* species, but rather either as a consequence of insect feeding or caused by other pathogens, was not recorded. The ear surface covered with mycelium was estimated in percentage according to an available scale [29]. The rotting area of single internodes was similarly estimated in the following percentage scales: 1–5%, 6–10%, 11–20%, 21–30%, 31–40%, 41–50%, 51–60%, 61–70%, 71–80%, 81–90%, 91–100%.

### 2.3. Isolation and Identification of *Fusarium* Species from Maize Particles

In order to assess the amount of viable propagules of fusaria, silage maize from each harvest sample was incubated on a Petri dish (9 cm diameter) with *Fusarium*-selective modified Nash-Snyder medium [30]. From the harvest material, 30 g of silage maize were washed under streaming water, dipped in a sieve into 1% ChloraminT solution (Riedel-de-Haën, Seelze, D) for 2 s, washed in sterile distilled water and distributed on cellulose cloth. Particles larger than 0.7 cm were selected by different fractions: 20 particles of maize kernels, leaves, husk leaves and stalks, respectively and 10 particles of both male florescences and rachis. A total of 200 particles in two replicates of 100 each were assessed for each sample. After 12 days of incubation in the dark at 19 °C, *Fusarium* colonies were transferred to potato dextrose agar (PDA, Oxoid, Hamphsire, UK) and nutrient low agar (“speziell nährstoff-armer Agar” [31]) containing a filter paper in 5.5 cm Petri dishes, respectively. Plates were subsequently incubated for 7 days at 19 °C with 12 h dark/12 h near-UV light. *Fusarium* species were identified based on macroscopic (mycelium shape and pigmentation) and microscopic characteristics (presence/absence and shape of macroconidia, microconidia, chlamydospores) [15]. The *Fusarium* incidence was calculated as mean of the number of isolates obtained from 100 particles silage maize from two replicates.

### 2.4. Toxin Analysis

The selected trichothecenes and zearalenone were quantified with a non-validated liquid chromatography tandem mass spectrometry (LC-MS/MS) method which was adapted from Dorn *et al.* [32]. 

Two and a half grams of ground silage maize were placed in a 100 mL flask and 25 mL of an acetonitrile/acetone/water mixture 50:25:25 (v:v:v) (acetonitrile and acetone from Scharlau Multisolvent, Sentmenat, E; water from Gradient A10, Millipore, Bedford, MA, USA) were added. Closed flasks were manually agitated until no more aggregates were visible and placed on a rotary shaker (Bühler SM-30, Hechingen, D) at 180 rpm. After two hours extraction time, the supernatant was decanted in a 15 mL vial with a solid screw cap (Supelco, Bellefonte, PA, USA) without filtration. Matrix components including chlorophyll, lipids or fat were reduced by cleaning 1 mL of extract over a 3 mL cartridge (Isolute, Uppsala, S) filled with 0.3 g of celite (Fluka, 545 coarse, Buchs, CH)/alox (Fluka, for chromatography, Buchs, CH) 1:1 (w:w), wetted and pre-cleaned with 2 mL of the same solvent mixture used for extraction. The resulting extract was collected in a 5 mL Reacti-vial (Supelco, Bellefonte, PA, USA). After percolation of 1 mL extract, the cartridge was rinsed with 2 mL solvent mixture and emptied by use of vacuum. The final volume of the cleaned extract (3.5 mL) was reduced at 40 °C to 0.4 mL with compressed air and transferred into a 2 mL HPLC-vial. The Reacti-vial was rinsed with 0.4 mL water/methanol 90:10 during 10 s by the aid of a vortex (Scientific Industries, Bohemia, NY, USA) and transferred to the HPLC-vial as well. The final volume of the extract was adjusted with water/methanol 90:10 to 1 mL. The samples were stored in the dark at room temperature and were processed within 48 h.

Liquid chromatography tandem mass spectrometry was performed on a 1200 L system (Varian Incorporation, Walnut Creek, CA, USA). The analytes DON, nivalenol (NIV), acetyl-deoxynivalenol (AcDON: sum of 3-AcDON and 15-AcDON), neosolaniol (NEO), diacetoxyscirpenol (DAS), HT-2 and T-2 toxin (all from r-biopharm, Glasgow, UK) as fusarenone-X (FUS-X) and ZON (Sigma-Aldrich, St. Louis, MO, USA) were separated on a Polaris C18-A column, (50 × 2.0 mm, 3 µm; Varian Incorporation, Walnut Creek, CA, USA). Operating the mass spectrometer in negative atmospheric pressure chemical ionization mode (APCI), the analytes NIV, DON, Fus-X, AcDON and ZON were detected with the next elution gradient: 0 min: 5% B (95% A); 1 min: 5% B; 4 min: 30% B; 5 min: 100% B; 12.5 min: 100% B; 13 min: 5% B; 20 min: 5% B. In positive APCI mode, the analytes NEO, DAS, HT-2 and T-2 were detected by applying the following gradient: 0 min: 20% B (80% A); 0.5 min: 45% B; 5.5 min: 75% B, 6 min: 100% B; 15 min: 100% B; 15.5 min: 20% B; 20 min: 20% B. The eluents were the same for both gradients. Eluent A consisted of water/methanol 95:5 (v:v), eluent B of water/methanol 5:95 (v:v). To enhance the formation of ions and adducts of certain analytes, both eluents contained 5 mM ammonium acetate (Fluka, puriss p.a., Buchs, CH). Each analyte was detected with two transitions (qualifier and quantifier) in multiple reaction monitoring (MRM). Analyte identification was confirmed using chromatographic retention time, correct mass of the mother ion, correct mass of the two daughter ions and agreement of the ratio of qualifier to quantifier with the calibration (±10%). All samples were quantified against pure standard calibrations. No correction for the matrix-dependent ion suppression was made. Therefore, the determined concentrations of the analytes in the samples must be interpreted cautiously.

Detection (quantification) limits were 20 (65) µg kg^−1^ for NIV, 78 (260) µg kg^−1^ for DON, 19 (64) µg kg^−1^ for FUS-X), 14 (46) µg kg^−1^ for AcDON, 20 (65) µg kg^−1^ for ZON, 4 (14) µg kg^−1^ for NEO, 2 (7) for DAS, 15 (50) µg kg^−1^ for HT-2 and 3 (10) µg kg^−1^ for T-2.

Fumonisins (total of B1, B2, B3) were measured by ELISA kits (Ridascreen^®^FAST Fumonisin, r-biopharm, D). 

### 2.5. Statistical Analysis

Means of the total incidence of *Fusarium* species, the five most prevailing species and DON concentrations were compared between different cropping factors by a one-way analysis of variance (ANOVA). For this, traits of cropping factors were grouped (hybrid: LG32.20, Amadeo, others; precrop and pre-precrop: cereals, silage maize, meadow, others; incidence of European corn borer (*Ostrinia nubilialis*): yes, no; soil cultivation: plough, none; harvest date: early (September); late (October); seed treatment: yes, no. Data were tested on normal distribution and equal variances and data analysis was performed with a level of significance of α = 0.5. If necessary, DON data were ln-transformed and infection rates were arcsine square root-transformed to achieve normal distribution. Differences between means were analyzed by a Holm-Sidak test on significance in order to find pairs of cropping factor and incidence of *Fusarium* species or DON content. The total amount of DON and the incidence of potential DON producers were correlated with calculation of Pearson’s correlation coefficient and the coefficient of determination (R^2^). The analyses described above were performed with SigmaStat^®^3.5 [33]. 

In order to find determining factors influencing toxin production, a multiple linear regression model, which was established by forward stepwise regression, was calculated using R 2.10.0 [34]. Normal distribution of DON over the sample sites was verified by plotting of log-transformed DON data. In order to minimize loss of information by transformation, untransformed DON data were analyzed in a generalized linear model, assumed to follow normal distribution (family = gaussian) with a link function suggesting log-distributed errors (link = log). The impact of cropping factors was estimated by calculating all possible regressions from one to four cropping factors as independent variables. If the type of a factor appeared only rarely in the data set, types were partly grouped in order to minimize the degrees of freedom. The grouping was carried out as in ANOVA analysis, except for the following factors: The date of the earliest harvest among the sample was set to zero and all subsequent harvest dates were counted in days from the earliest. Seed treatment was set to yes/no. Seed bed preparation remained without grouping, because a grouping did not improve the respective model. The obtained regression models were evaluated by the small-sample Akaike Information Criterion (AIC_c_) [35], with the R-package “AICcmodavg” [36]. R^2^ was calculated as the ratio of explained variance and the total variance. 

## 3. Results and Discussion

### 3.1. *Fusarium* Species Spectrum on Silage Maize and Power of Prediction of Infection by the Pre-Harvest Disease Rating

Overall, few disease symptoms were observed on maize ears and stalks in the 22 fields, where a pre-harvest disease rating was conducted. Out of a sum of 1100 ears over all sample sites, only 61 ears were infected (data not shown). From these ears, 43 were visibly infested on 1–3% of the surface. At seven sample sites, no infected ears were recorded and at four sample sites, no infected stalks were recorded. Among these, one sample site showed neither ear nor stalk rot. From all internodes (*n* = 5535) over all sample sites, only 1.7% were infected compared with 5.5% of all ears. 

**Figure 2:**
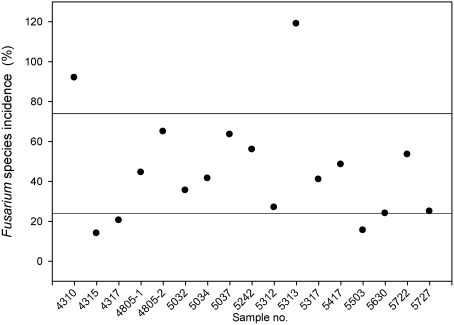
Total *Fusarium* species incidence of each silage maize sample (average of two replicates). Seventeen of 19 harvest samples were analyzed by the morphological plating-technique (one of the remaining samples was mature silage and the other the other one did not result in *Fusarium* isolates). More than 100% *Fusarium* incidence can result, because more than one isolate can grow from one particle.

Based on the analysis by plating maize particles on agar, two-thirds of the 17 harvest samples were infected and infection ranged between 25% and 75% total *Fusarium* species incidence (Figure 2). The average *Fusarium* incidence was 46%. This finding did not agree with the observed disease symptoms before harvest. The number of *Fusarium* isolates out of 200 particles of silage maize ranged between 28 isolates (site 4315) and 238 isolates (5313). Since one particle can lead to several *Fusarium* isolates, the infection rate (mean of both replicates) can exceed 100%.

Over all sample sites, 12 different *Fusarium* species could be identified (Table 1). The most prevalent species were *F. sporotrichioides*, *F. verticillioides* and *F. graminearum *(each with 16%)(Table 1). *Fusarium avenaceum*, *F. proliferatum* and *F. equiseti* were also frequently identified. The abundance of individual species varied among the sample sites. For example, *F. graminearum* clearly dominated in sample 5722, while *F. avenaceum* was the prevalent species in sample 5313. The number of different species per sample ranged from five (5630) to eleven (5037). *Fusarium verticillioides* and *F. graminearum* were detected in every sample analyzed, *F. proliferatum* and *F. sporotrichioides* in 16, *F. avenaceum* in 15 and *F. crookwellense* in 14 samples. *Fusarium equiseti*, *F. oxysporum*, *F. poae* and *F. subglutinans* were observed in ten to twelve samples and *F. culmorum and F. tricinctum* were found in five and six samples, respectively.

Plating harvest material on agar revealed a considerable infection of the plants by *Fusarium* species, although this could not be assumed by the pre-harvest disease rating. The phenomenon of symptomless infection by *Fusarium* species is in accordance with previous findings [11]. The most important reason for the discrepancy between visual pre-harvest symptoms and real infection is supposed to be the symptomless endophytic growth of some *Fusarium* species. *Fusarium verticillioides*, one of the prevailing species in this study, is reported to grow systemically within the plant without causing any disease symptoms [37,38]. Another possible reason for these contrasting results between field and laboratory assessment might be the fact that the disease ratings on different sites were performed in a range from the harvest date itself up to seven weeks before harvest. It was not possible to conduct all ratings close to the harvest since most growers did not determine their individual harvest dates in advance. Further, the time of the year plays an important role in the development of disease symptoms. Grain maize, which is harvested later than silage maize, allows at least the identification of highly infected fields, because there is more time for developing symptoms, although the prediction of disease and mycotoxin contamination is also very limited [39,40]. Another aspect is the type of rot rating. Non-fusarial rot and fusarial rot caused by wounds from animal feeding cannot be distinguished in the field. Therefore, only rot on ears and internodes, which was found not to be a consequence of feeding by pests [24] and birds [41], was recorded. In this study, the incidence of the European corn borer during disease rating was low (average: 3.8). In summary, the pre-harvest disease rating of natural *Fusarium* infection on visual *Fusarium* symptoms is not suitable to predict the real *Fusarium* infection or the possible toxin content of silage maize. This is in contrast to studies using artificial inoculations with a specific *Fusarium* species, which found a positive correlation between visual disease assessment and DON contamination of maize ears [42]. We suppose this discrepancy is due to different conditions, mainly artificial silk channel infection with one species *versus* natural infection by various species through many different means.

**Table 1:** Number of isolates of individual *Fusarium* species obtained from 17 harvest samples of silage maize.

Sample no. (postal codes)	F. sporotrichioides	F. verticillioides	F. graminearum	F. avenaceum	F. proliferatum	F. equiseti	F. poae	F. oxysporum	F. crookwellense	F. subglutinans	F. culmorum	F. tricinctum	F. spp.^1^	Total
4310	51	24	7	3	42	28	1	15	3	4	0	0	6	184
4315	1	6	4	6	3	0	3	2	1	0	0	0	2	28
4317	6	4	1	7	1	14	0	0	6	0	0	0	2	41
4805-1	12	24	2	1	1	25	1	13	5	0	0	0	5	89
4805-2	19	8	18	27	9	18	14	3	5	0	1	1	7	130
5032	27	2	13	15	3	1	1	2	1	3	0	1	2	71
5034	31	16	1	0	29	1	0	1	0	0	0	0	4	83
5037	20	21	23	5	21	10	3	7	2	1	2	0	12	127
5242	19	10	25	13	4	16	6	1	2	1	0	0	15	112
5312	6	7	6	6	3	10	2	0	4	2	0	0	8	54
5313	18	47	26	40	29	6	32	1	8	4	0	1	26	238
5317	4	9	36	16	0	5	0	0	0	2	2	0	8	82
5417	16	22	15	7	8	1	1	0	8	3	1	1	14	97
5503	5	4	3	11	1	0	0	0	2	3	0	2	0	31
5630	0	39	4	0	2	0	0	1	0	1	0	0	1	48
5722	8	4	53	20	3	0	4	0	1	0	2	1	11	107
5727	14	6	8	2	12	0	0	1	1	0	0	0	6	50
**Total**	257	253	245	179	171	135	68	47	49	24	8	7	129	1572
**%**	16.3	16.1	15.6	11.4	10.9	8.6	4.3	3.0	3.1	1.5	0.5	0.4	8.2	100
**Mean**	15.1	14.9	14.4	10.5	10.1	7.9	4.0	2.8	2.9	1.4	0.5	0.4	7.6	92.5
**SEM^2^**	3.7	3.6	3.5	2.6	2.4	1.9	1.0	0.7	0.7	0.3	0.1	0.1	1.8	22.4
**Rank**	1	2	3	4	5	6	8	9	9	10	11	12	7	

^1^ not identified to species level. ^2^ SEM = standard error of mean.

The spectrum of fusaria on silage maize with 12 different species confirms recent findings of surveys on maize kernels and stalks in Switzerland [8], Belgium [11] and on kernels in Germany [13] and emphasizes the complexity of *Fusarium* species in maize compared with wheat, where five species mainly contribute to the disease (reviewed in [2]). The dominating species in this study, *F. sporotrichioides*, *F. verticillioides*, *F. graminearum*, *F. avenaceum* and *F. proliferatum* are common maize pathogens [15]. From these prevailing species, *F. graminearum* is the most prevalent species, and *F. avenaceum* is a frequently associated species of FHB in wheat in central and western Europe [2,43], whereas *F. sporotrichioides* is occasionally observed in maize, but not with a high incidence [8,11,13]. In this survey, however, *F. sporotrichioides* was the dominant species occurring with 16.3%. Additionally, it occurred in 16 out of 17 sample sites and thus, it was as widely distributed as *F. verticillioides*, *F. graminearum*, *F. proliferatum* and *F. avenaceum*. Although *F. sporotrichioides* was detected in Germany in 30% of all maize kernel samples in 2006, the average number of *F. sporotrichioides* infected kernels per sample site was less than 2% [13]. Furthermore, a harvest monitoring of field samples from different small-grain cereals in 2010 in Bavaria, Germany, revealed an unexpected incidence of *F. sporotrichioides* [44]. The high incidence of *F. sporotrichioides* might be a part of annual variation or due to the small sample size, but nevertheless, as a producer of the highly toxic trichothecenes T-2 and HT-2, the occurrence of *F. sporotrichioides* should be addressed in further investigations.

**Figure 3:**
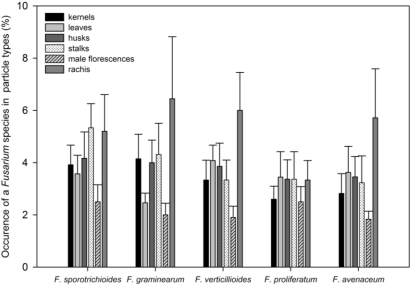
Distribution of the five most prevalent *Fusarium* species in different particle types (means and standard errors of means). Infection rates of rachis and male florescence were doubled for the diagram, because half the amount of these particle types were planted.

There was no marked specificity in colonization of individual particle types, neither for the total amount of all *Fusarium* species, nor for individual *Fusarium* species (data not shown). However, among the prevailing species there was a trend that *F. graminearum* occurred less in leaves compared with other species. Furthermore, *F. proliferatum* appeared to grow more frequently in male florescences compared with other species, but not more often than in other particle types (Figure 3). *Fusarium culmorum* was not found in leaves and the rachis, while *F. tricinctum* could not be isolated from leaves, rachis and male florescences. This finding is possibly due to the generally low incidence of these two species.

### 3.2. Toxin Spectra and Correlations between *Fusarium* Species and Toxins

DON was detected in every sample and ranged from 0.78 to 2.99 mg kg^−1^. Thirteen out of 19 samples exceeded the EU and Swiss guidance value for maize-based supplementary and complete feeding stuffs for swine (0.9 mg kg^−1^) [18], a highly susceptible animal species. In four samples, the DON guidance value of 2 mg kg^−1^ for calves feeding stuffs was exceeded. With respect to maize byproducts for animal feed raw materials (12 and 3 mg kg^−1^, respectively) [18], all DON and ZON values (Table 2) were below the EU guidance values. Zearalenone was found in 15 samples with a maximum level of 430 µg kg^−1^, which is close to the guidance values of 0.5 mg kg^−1^ for dairy cows’ feedstuffs. Further, ZON exceeded the guidance value for supplementary and complete feeding stuffs for adult swine (250 µg kg^−1^) in three samples and for young pigs (100 µg kg^−1^) in five samples. Five ZON values ranged between the detection and the quantification limit of 20–65 µg kg^−1^ (Table 2). Nivalenol was detected in eight samples ranging between 190 and 760 µg kg^−1^. T-2 and HT-2 toxin occurred rarely with maximum values of 130 and 84 µg kg^−1^, respectively. AcDON was very rare and NEO, DAS and FUSX were not detected at all. Fumonisins were detected in one sample below the guidance value. In summary, because of contaminations by DON and ZON, 13 of 19 samples were not suitable as supplementary and complete feedstuff for adult swine, though maize silage feeding of swine is rather seldom. However, in this study silage maize samples revealed a large spectrum of *Fusarium* mycotoxins and possible additive effects, as assumed for many trichothecenes, might pose a risk for animal health that needs to be examined [45]. 

**Figure 4:**
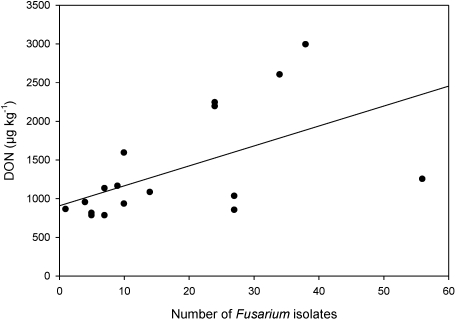
Correlation between the number of *Fusarium* species potentially producing deoxynivalenol (DON) (sum of *F. graminearum*, *F. culmorum* and *F. crookwellense* obtained from 200 particles of silage maize) and measured DON content.

The correlation between DON and the incidence of potential DON producers (*F. graminearum*, *F. culmorum*, *F. crookwellense*) with a determination coefficient of 0.31 was weak (Figure 4). In contrast, the determination coefficient from a wheat monitoring in Switzerland was 0.71 [43]. The present study was conducted with a much smaller sample size (*n* = 19, compared with *n* = 248 [43]) and deals with maize, which is host of many more different *Fusarium* species than wheat. In fact, inter-species interactions play an important role in toxin production. In a study with artificial silk channel infections with either *F. graminearum*, *F. verticillioides* or a mixture of both, it was demonstrated that *F. graminearum* alone produced greater disease symptoms and amounts of ergosterol, followed by the mixture and than by *F. verticillioides* alone [14]. Furthermore, the inhibiting effects of *F. subglutinans* on the DON production by *F. graminearum* were observed in an *in vitro* study [46]. One sample was not included in the scatter plot, since could not be analyzed by the plating-technique: DON was detected, although no isolates of DON producers were found. Many non-fusarial fungi were found in this sample and could have inhibited the growth of *Fusarium* species, of which only two isolates could be obtained. 

**Table 2:** Concentration of trichothecenes and ZON detected in 19 samples of silage maize.

Toxin(µg kg^−1^)												
Sample NO.	DON		NIV		AcDON		HT-2			T-2		ZON
4310	930		190		nd		72			26		d
4315	780		200		nd		nd			d		d
4317	1130		560		d		nd			40		nd
4805-1	780		380		nd		130			42		97
4805-2	2190		nd		nd		120			84		430
5032	**1080**		700		nd		76			31		88
5034	860		690		nd		nd			14		d
5037	850		nd		nd		nd			16		94
5224	900		nd		nd		76			nd		d
5242	**1030**		690		nd		nd			nd		nd
5312	1590		nd		nd		nd			nd		nd
5313	2600		nd		d		nd			nd		d
5317	**2990**		760		135		nd			nd		260
5417	2240		nd		300		nd			nd		280
5503	810		nd		nd		nd			nd		150
5604	1650		nd		nd		nd			nd		230
5630	950		nd		nd		nd			nd		nd
5722	1250		nd		nd		nd			nd		83
5727	1160		nd		nd		nd			nd		97
**Mean**	1356		521.3		217.5		94.8			36.14		180.9
**EU Guidance Level**	900 ^1^–**12000**^2^		n.a.		n.a.		n.a.			n.a.		**100**^1^–3000 ^2^

^1^ Lowest guidance value, depending on animal species, type of feedstuff and animal age. ^2^ Guidance value for maize byproducts as animal feed raw materials [18]. nd = not detected; d = detected, but below quantification limit; n.a. = not available.

Although NIV was found in only eight samples, more attention should be paid to this toxin, because of its seventeen-fold higher toxicity in mice (LD_50_ = 4.1 mg kg^−1^) compared with DON (LD_50_ = 70 mg kg^−1^) [47]. Concerning T-2 and HT-2, a higher contamination by these toxins could have been expected, due to the high incidence of *F. sporotrichioides*, which is able to produce these toxins. The largest amounts of 130 and 120 µg kg^−1^ HT-2 and 84 and 42 µg kg^−1^ T-2, respectively, were found in Brittnau (samples 4805-1 and 4805-2), but conditions which may have favored the toxin production here are not known. Despite the high toxicity of these two toxins (LD_50_ in mice are 5.2 mg kg^−1^ (T-2) and 9.2 mg kg^−1^ (HT-2) [47]), guidance or limiting values for feed or food do not yet exist. Although *F. sporotrichioides* is able to build NEO and DAS, these toxins were not detected throughout this study. This might again be due to fungal interactions between the numerous *Fusarium* species in silage maize [14,46]. Another important point, which is relevant for all toxin measurements, is that real toxin contents might be higher due to so called “masked mycotoxins”. These are soluble conjugates of toxins which are built by chemical transformation processes (e.g., during detoxification) by the plant, other microbes, the producing fungus itself or by further food processing like heating [48]. Bound conjugates also exist, which become, e.g., part of the cell wall [48]. This phenomenon could be problematic since toxic effects of such conjugates are widely unknown [48]. Conjugates might be retransformed into the parent toxin by digestion or metabolic transformation and contribute to the entire toxin content without being measured as already described for ZON [49]. Masked mycotoxins might be measured, but not discriminated from the analytical target toxin, as it is the case for AcDON in DON-ELISA measurements. From the toxins that were investigated in this study, conjugates are known for DON, ZON, NEO, T-2 and FUM [48].

### 3.3. Prediction of DON Content by Cropping Factors with a Regression Model

Samples with high DON content were often from fields harvested after September (*n* = 10). Cropping factors increasing the risk of DON production could not be identified by ANOVA analysis, although a harvest date after September 30th tended to result in a higher DON content (data not shown). In order to find other strong impacts of cropping factors, a generalized linear regression model was built in a forward selection mode. The model with the best quality, assigned by the lowest AIC_c_, included the three cropping factors “harvest date”, “pre-precrop” and “seed treatment” (Table 3). Here, DON and the three factors correlated with *R*^2^ = 0.61. Between harvest date and pre-precrop, an interaction was observed. Since the harvest date of the present crop should not be influenced by a crop two seasons before, this interaction is most probably due to coincidence. Sample 5417 with the highest DON concentration of almost 3 mg kg^−1^ strongly influenced the model. A calculation without this sample revealed its leverage effect by a strongly decreased *R*^2^ (0.44). As of yet, a possible explanation for this well above-average DON value is missing. If additional cropping factors or factors with more levels were included into the regression model, *R*^2^ continued to increase, but the regression model became over-specified leading to an increasing AIC_c_ value. 

**Table 3:** Establishment of a generalized linear model.

Step	Factors				AIC_c_	R^2^
1	Harvest date				302.2	0.26
2	Harvest date	Hybrid			308.0	0.31
	Harvest date	*O. nubilalis*			299.8	0.45
	Harvest date	Precrop			308.2	0.44
	Harvest date	Pre-precrop			285.8	0.55
	Harvest date	Soil cultivation			305.1	0.27
	Harvest date	Seed bed Prep.			325.3	0.50
3	Harvest date	Pre-precrop	Hybrid		295.9	0.56
	Harvest date	Pre-precrop	*O. nubilalis*		285.6	0.66
	Harvest date	Pre-precrop	Precrop		301.9	0.58
	Harvest date	Pre-precrop	Soil cultivation		286.2	0.65
	Harvest date	Pre-precrop	Seed bed Prep.		328.0	0.73
	**Harvest date**	**Pre-precrop**	**Seed treatm.**		**274.6**	**0.61**
4	Harvest date	Pre-precrop	Seed treatm.	Hybrid	286.5	0.64
	Harvest date	Pre-precrop	Seed treatm.	*O. nubilalis*	277.9	0.67
	Harvest date	Pre-precrop	Seed treatm.	Precrop	281.7	0.64
	Harvest date	Pre-precrop	Seed treatm.	Soil cultivation	275.4	0.71
	Harvest date	Pre-precrop	Seed treatm.	Seed bed prep.	346.2	0.85

The best model showing the lowest AIC_c_ is in bold letters. Hybrid = maize hybrid, prep. = preparation, treatm. = treatment, *O. nubilalis* = *Ostrinia nubilalis* (European corn borer).

It is already known that a late sowing date, and consequently a late harvest date, increases the risk of DON contamination [23], possibly due to the longer time period for growth and toxin production for *Fusarium* species. Effects of precrops on the incidence and potential toxin production in wheat have been described in several studies [22,25,26]. Interestingly, in the present regression model, the pre-precrop contributed as a prediction factor, while the precrop did not. This result confirms previous investigations demonstrating the ability of *Fusarium* species to survive for longer than one year on crop residues in the soil [50]. One possible reason for this finding is the fact that, after harvest of the precrop, growers with tillage cropping systems plough crop residues into the soil. Simultaneously, they move up non-decomposed residues of the pre-precrop onto the soil surface, which might serve as inoculum source for the cultivar. However, eight of the 19 fields in this study were cultivated with a catch crop, hence the growers already might have moved up the pre-precrop residues onto the surface before the sowing of the catch crop. Alternatively, *Fusarium* species might survive on alternate hosts on or beside the field, which was hypothesized for *F. verticillioides* on grass in this study (data not shown) and which could explain the surprisingly strong impact of the pre-precrop. 

The third cropping factor of the regression model was the seed treatment. Most of the growers used treated seeds and most of them applied the product Mesurol^®^ (active ingredient: 500 g/L Methiocarb). Mesurol^®^ is efficacious against some insects and acts as a repellent against birds, but it is not a fungicide. According to Bayer CropScience [51], a reduction of infection by the fungal pathogen *Ustilago maydis* should be achieved by reducing feeding wounds through insects and birds. This may also be the case for infection by *Fusarium* species, for which it is known that animals may serve as vectors [24,39,41]. 

Seed bed preparation was not included in the final model, although it provided a high *R*^2^ as third cropping factor among the three-factor models. This model was discarded, because it had the highest AIC_c_. An explanation for that might be the high number of different levels (types of seed bed preparation methods) of this factor, which increases the degrees of freedom and thus increases the AIC_c_. Even different ways of grouping the various types of seed bed preparation methods did not result in a lower AIC_c_ and none of these levels were significant.

In the current study, factors, which were expected as relevant cropping factors, mainly soil cultivation and precrop, did not contribute to the model. This is probably due to this data set: A combination of cereals or silage maize with no-tillage cropping, representing a higher risk for infection by *F. graminearum* and DON contamination, occurred only twice. These samples had the precrop barley and wheat and each contained DON values around the average of this study (1.1 and 1.6 mg kg^−1^). Overall, care should be taken when interpreting the regression model results due to the limited sample size.

**Figure 5:**
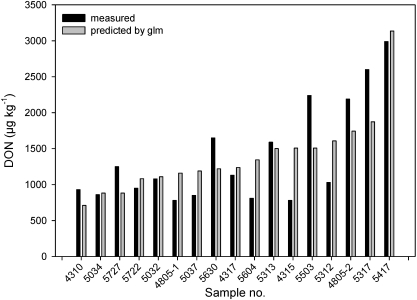
Comparison of predicted and measured DON contents in silage maize samples based on the generalized linear regression model.

A comparison of the measured and the predicted DON data by the regression model revealed that nine of the predicted DON values showed a relatively high deviance with more than 25% from the measured DON value (Figure 5). From those, five samples contained less DON than predicted, but all showed a lower *F. graminearum* incidence than the average, which is probably the main reason for this finding for *F. graminearum* is the main DON producer. Further, four of the five samples containing less DON than predicted came from a ploughed field with a presumably less susceptible hybrid and no sign of European corn borer infection, which are all factors supposed to reduce infection. The four samples, where measured DON contents were higher than the predicted ones, could be explained in one case by an above-average incidence of *F. graminearum*. 

This model is based on a rather small sample size and was manually chosen by forward selection. Models with a low number of dependent variables and many potentially explaining factors run a higher risk of choosing factors as variables, which appear significant, but have no causal relationship. This model selection bias is also known as Freedman’s paradox [52]. An automated model selection based on model averaging and an information criterion as the AIC_c_ was recently implemented as an “R”-package (“glmulti” [53]) and could overcome this problem.

## 4. Conclusions

In this study on the *Fusarium* species in silage maize samples, twelve different *Fusarium* species were identified, of which *F. sporotrichioides*, *F. verticillioides*, *F. graminearum*, *F. avenaceum* and *F. proliferatum* where the most prevalent species. All 19 samples contained the trichothecene DON, partly exceeding European and Swiss guidance values for animal feed, which emphasizes the relevance of our research. Furthermore, ZON, other trichothecenes such as nivalenol, HT-2 and T-2, and acetylated DON as well as FUM were found. In order to explain contamination with DON by cropping factors, a generalized linear regression model was established containing the cropping factors harvest date, pre-precrop and seed treatment. Especially the role of a late harvest on toxin contamination was demonstrated by this study. In contrast, the influence of tillage practice and precrop could not be confirmed, which is probably due to the low number of samples in the data set. Our investigation of *Fusarium* species and mycotoxin contamination of silage maize indicate that maize silage, which is not traded and therefore not controlled for toxin contamination, might pose a risk to animal health. We suggest conducting a European-wide monitoring of silage maize to identify the environmental and cropping factors influencing infection by *Fusarium* species and contamination by trichothecenes and other mycotoxins of this important animal feed. A better understanding of such factors and the interaction between the toxigenic species could contribute to reducing the potential risk of this feed to animal health. 
